# Development and Effects of *Schistosoma japonicum* (Trematoda) on its Intermediate Host, *Oncomelania hupensis* (Gastropoda)

**Published:** 2013

**Authors:** Y Sulieman, T Pengsakul, Y Guo

**Affiliations:** Parasitology Research Laboratory, School of Life Science, Xiamen University, Xiamen 361005, Fujian Province, People's Republic of China

**Keywords:** *Oncomelania hupensis*, *Schistosoma japonicum*, Miracidia effect, Cercariae rhythm

## Abstract

**Background:**

Trematodes belonging to the genus *Schistosoma* cause schistosomiasis. The relationship between schistosomes and their intermediate hosts varies among snails. This study investigated the effects of *S. japonicum* on its snail host, *Oncomelania hupensis*, and cercarial release rythmicity of *S. japonicum* and the effects of light on it.

**Methods:**

Seven groups of *O. hupensis* (n = 40 each) were exposed individually to 0 (control), 2, 4, 6, 10, 15, and 20 *S. japonicum* miracidia. Mortality of the snails was recorded for 10 weeks. Snails in each group were checked for infection at seven weeks post-exposure. Positive snails were exposed to artificial light from 06:00 am – 18:00 pm and the liberated cercariae were collected every 2 hours to determine the rhythmicity of cercarial release. Three groups of positive snails (n = 6 each) were exposed to artificial light, daylight, and darkness from 06:00 am – 18:00 pm, the liberated cercariae were collected every 2 hours to determine the effects of light.

**Results:**

The highest infection rate and host mortality occurred among snails in the groups exposed to 15 and 20 miracidia. Cercariae were liberated after eight weeks of exposure of *O. hupensis* to *S. japonicum*. The circarial emerging pattern was circadian, with a single peak of emerging between 10:00 am and 12:00 pm. Light intensity had a positive influence on cercariae shedding and rhythmicity.

**Conclusion:**

Further research, including the influence of biotic and abiotic factors is deemed necessary to fine-tune elucidation of the effects of *S. japonicum* upon *O. hupensis* snail.

## Introduction

Schistosomiasis is one of the major communicable diseases of public health importance in tropical and subtropical regions of developing countries (1, 2). Schistosomes of the genus *Schistosoma* are the causative agents of the disease and are transmitted to the mammalian definitive hosts by freshwater gastropod (Gastropoda) snails, e.g., the amphibious snail, *Oncomelania hupensis* which transmits *Schistosoma japonicum*, as well as the truly aquatic snails, such as *Biomphalaria* sp. and *Bulinus* sp., which transmit *S. mansoni* and *S. haematobium*, respectively, to humans (3). Schistosomes use these snails as first intermediate hosts, where several larval stages generally develop within the snails, including sporocysts, mother sporocysts and cercariae (4, 5).

Trematode infections have a negative effect on snails *e.g*., mechanical cell damage inflicted due to migration of the larval stages of trematodes, consumption of the digested food materials, secretion of toxic substances by the parasite, and the host reproductive success limitation (6, 7); these negative effects may vary from one snail host to another.

Production of cercariae varies widely among different human schistosome parasites, and is related to the size of the intermediate host to some degree (8), and the host-parasite compatibility, i.e., high level of susceptibility of the snail results in high cercarial productivity (9). The rate of cercarial production is generally not proportional to the miracidial dose received by the snails, although in some host-parasite combinations, productivity of polymiracidial infections is shown to be higher than that of monomiracidial infection and vice versa (4, 10). Each sch-istosome cercaria species has a unique daily rhythm of leaving the intermediate-hosts; these rhythms are either circadian (i.e., one emergence peak/24 h), or more, but rarely ultradian or bimodal (i.e., two emission peaks/24 h). Photoperiod and thermoperiod are the most important factors affecting shedding patterns of cercariae; among these, light is the principal stimulus that causes emergence of both *S. haematobium* and *S. mansoni* cercariae, thus, shedding of cercariae may take place at water temperatures between 10 to 30 °C, or even higher (11).

In the present study, effects of *S. japonicum* miracidia infection at different treatment rates upon the host snail, *O. hupensis*, were investigated, and the cercarial shedding patterns as well as the effects of light on cercarial shedding patterns were studied.

## Materials and Methods

### Field collection of O. hupensis and harvesting of miracidia

*Oncomelania hupensis* were collected in May 2011, from the western part of the Dongting Lake, Hunan Province, China, and were transported to the Parasitology Research Laboratory, School of Life Science, Xiamen University. The collected snails were screened individually three times at an interval of seven days for any patent infection. Non-infected snails were separated and were maintained in the laboratory (photoperiod: 12h light/12h dark; room temperature: 25 ± 1°C) in 30 x 20 x 5 cm containers that were paved with wetted filter papers. These laboratory-maintained snails were fed every three days on wheat flour mixed with water. *Schistosoma japonicum* eggs were obtained by homogenizing (using a blender) laboratory- infected livers of white mice in 0.9% normal saline solution. The mixture was passed through a series of four stainless steel sieves with decreasing pore size of 300, 150, 100, and 40 µm The column was washed repeatedly with normal saline and the trapped *S. japonicum* eggs on the surface of the finest pore sieve were transferred with a Pasteur pipette to Petri-dishes containing clear de-chlorinated tap water, and placed under a fluorescent light for 2 to3 hours to stimulate hatching of miracidia.

### Experimental design

The laboratory maintained healthy *O hupensis* (shell length 6 to 8 mm) were divided into seven groups with each group containing 40 individuals used in this study. Snails in each group were individually placed into a tissue culture plate chamber containing 2 ml of clear de-chlorinated tap water just sufficient to cover the snail. Snails in groups 1, 2, 3, 4, 5, 6, and 7 were exposed individually to 0 (control), 2, 4, 6, 10, 15, and 20 active *S. japonicum* miracidia, respectively, using a dissecting microscope, and a Pasteur pipette for picking the emerged miracidia from Petri-dishes. After the exposure, the plates were covered and were left overnight under laboratory light and temperature conditions to facilitate miracidia penetration. The following day, snails in each group were placed into 30 x 20 x 5 cm separate containers (one for each group) paved with wetted filter papers and kept under laboratory conditions. These snails were fed every three days on wheat flour mixed with water. Mortality of snails in each group was recorded every week. Screening for infected snails was started at the end of the seven week post-exposure to the miracadia. Dead snails were crushed immediately and were examined for infection and collected data were tabulated.

### Cercariae shedding rhythm

By the end of 11 weeks post-exposure, 18 positive (laboratory-infected) and three naturally-infected snails were collected. These snails were individually placed into a tissue culture plate chamber containing clear de-chlorinated tap water to its half depth and then covered and left under fluorescent light from 06:00 am to 18:00 pm. Snails were transferred to new chambers at every 2-hour interval. The harvested cercariae every 2-hour from each snail were killed by adding a few drops of Lugol's iodine solution and the settled cercariae were counted under a dissecting microscope. The experiment was repeated three times at an interval of five days.

### Effects of illumination on shedding of cercariae

Three groups (n = 6 per group) of positive snails were used in this experiment. These snails were placed individually into a tissue culture plate chamber containing 2 ml of clear de-chlorinated tap water, covered, and were placed over a holder into a water-bath, with the upper part of the plates slightly above the water surface. The water temperature in the water-bath was maintained at 29 ± 1°C. Group 1 snails were placed under normal daylight (near a window); group 2 was placed under a 10-watt electric lamp at a distance of 50 cm from the light source, and group 3 was placed in a dark room. Exposed snails were transferred into new plate chambers after every 2-hours, from 06:00 am to 18:00 pm. Number of cercariae emerging from snails in each group was counted. The experiment was repeated three times at an interval of five days.

### Data analysis

Paired comparisons were performed using a student's *t*-test, while multiple comparisons were analyzed using One-way ANOVA. An SPSS 16.0 statistical package (12) was used for the statistical analysis.

## Results

Out of 240 *O. hupensis* that were exposed to different treatment rates of *S. japonicum* miracidia, 190 (79.2%) remained alive by the end of seven weeks post-exposure, compared to 38/40 (95%) in the unexposed control group. At the termination of experiment at 10 weeks post-exposure, out of 240 exposed snails, 114 (47.5%) remained alive compared to 36/40 (90%) in the control group. Difference in mortality values between the exposed groups and control group of experimental snails was statistically significant (*P* < 0.05).

**Fig. 1:**
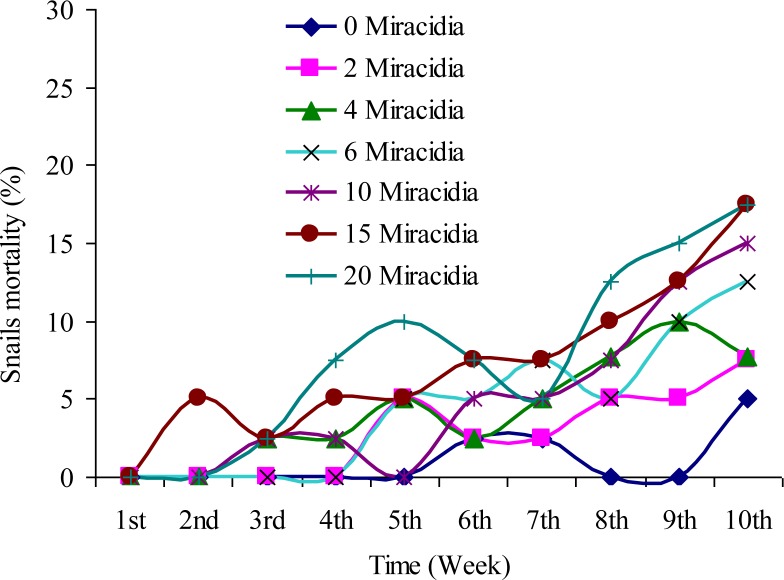
Post-exposure mortality among seven groups of *Oncomelania hupensis* snails exposed to different treatment rates of *S. japonicum* miracidia for 10 weeks

Highest rates of mortality of the snails, 72.5% and 77.5% as a total were recorded at the end of 10 weeks post-exposure among snail groups that were exposed to the treatment rates of 15 and 20 miracidia, respectively (Fig. 1). The mortality difference between the exposed groups of snails was statistically significant (*P* < 0.05).

**Fig. 2:**
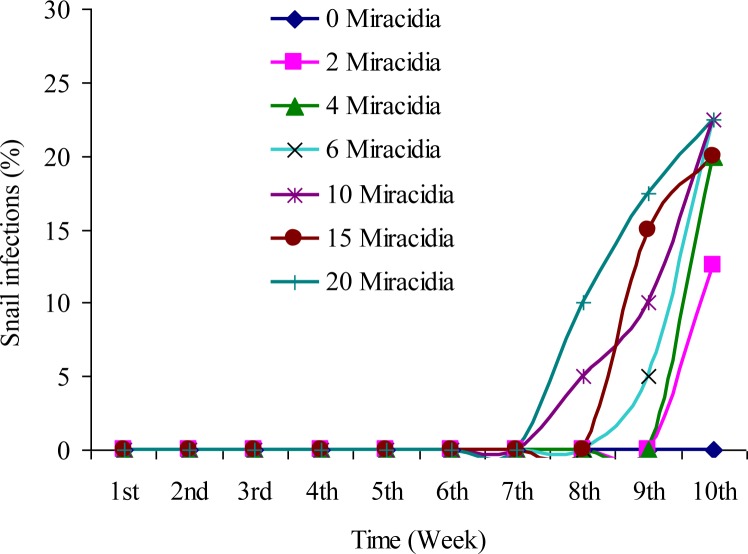
Post-exposure infection of *Schistosoma japonicum* among seven groups of *Oncomelania hupensis* snails exposed for 10 weeks to different treatment rates of *S. japonicum* miracidia

All *O. hupensis* snail groups that were exposed to a different treatment rate of *S. japonicum* miracidia were found free from infection at the end of seven weeks post-exposure, with a few snails found to acquire infection at the end of eight weeks post-exposure, i.e., 5% and 10% from groups 5 and 7 that were exposed to 10 and 20 miracidia, respectively. In subsequent two weeks, the infection rate among snails in groups 2 to 6, increased gradually with the increasing treatment rate of miracidia (Fig. 2).

**Fig. 3:**
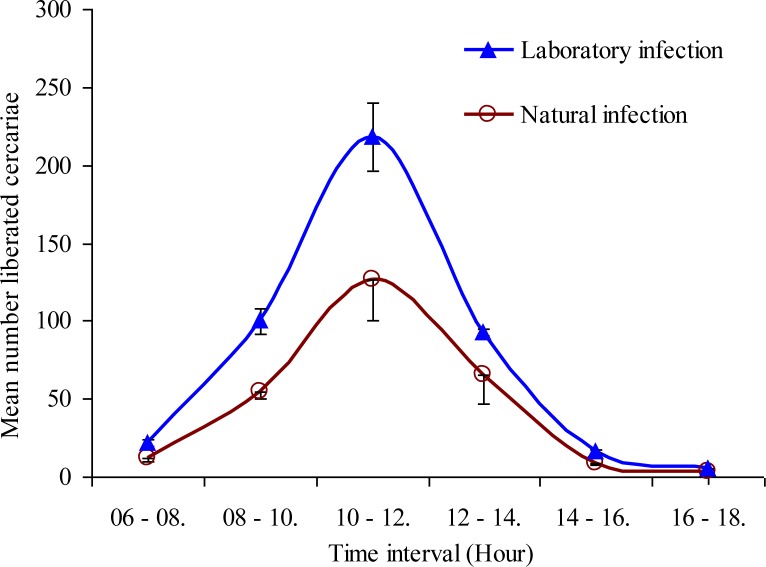
Pattern of *Schistosoma japonicum* cercariae liberation at 2-hour interval from laboratory-infected and naturally-infected *Oncomelania hupensis* snail, exposed to artificial light from 06:00 am to 18:00 pm

The emergence of *S. japonicum* cercariae from laboratory as well as naturally infected *O. hupensis* was found to follow a circadian pattern. Cercariae were noted to start emerging from *O. hupensis* two hours after the onset of the experiment at 06:00 am and achieved a single peak of emergence between the hours of 10:00 am to12:00 pm, declining gradually thereafter to minimum emergence at 18:00 pm (Fig. 3). The differences in the amplitude of emission rhythms of cercaria from the host during the monitored intervals were statistically significant (*P* < 0.05).

**Fig. 4:**
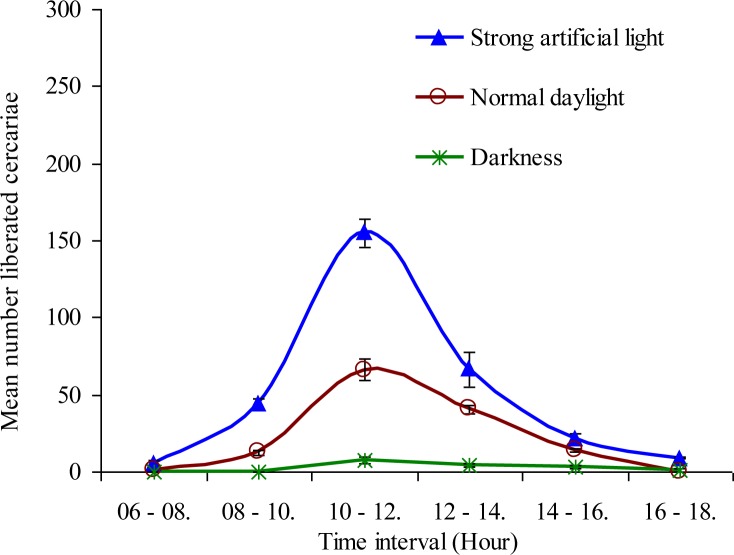
Mean number of *Schistosoma japonicum* cercariae liberated at an interval of 2 hours from three groups of laboratory-infected *Oncomelania hupensis* snails exposed to artificial light, normal daylight, and darkness from 06:00 am to 18:00 pm

The infected *O. hupensis* snails that were kept from 06:00 am to 18:00 pm under artificial light and room temperature of 29 ± 1°C were found to shed cercariae after 2 hours of the onset of experiment, with a continuous increase in shredding of cercariae, reaching a peak within a period of six hours and thereafter gradually declining in numbers until no shredding by 18:00 pm. Similarly, in another group of infected snails kept under normal daylight, the cercariae shedding pattern was similar to the first group, but the number of emerged cercariae was relatively low. On the other hand, when infected snails were kept under total darkness; shedding of cercariae was observed to start after nearly 4 hours of onset of the experiment, and very low numbers of cercariae emerged, without having any clear pattern of emergence (Fig. 4). The effect of light on the cercarial shedding patterns occurring between artificial light and normal daylight was statistically significant (*P* < 0.05).

## Discussion

In-depth knowledge of the effects of *S japonicum* miracidia infection upon snail hosts and the production dynamics of cercariae is crucial for understanding the schistosomes’ transmission strategies. The present study revealed that rates of survival of *O. hupensis* infected with *S. japonicum* were lower than the uninfected ones, and the highest levels of *O. hupensis* infection and mortality were observed among snails that were exposed to a high treatment rate of *S. japonicum* miracidia. High mortality among these snails may have been due to the high parasite load, the parasite's developmental activity and its location within the host. It has been known that the digenean flukes are important in terms of regulating populations of the host snails (13, 14) by increasing mortality of the infected host snails, especially under highly variable environmental conditions (15–17). The present research investigation revealed that *O. hupensis* exposed to 10 miracidia per snail survived longer and produced a reasonable number of cercariae. It was also observed that at 10 weeks post-exposure, production of cercariae was rather high among the different groups of exposed snails, unlike the observation at eight weeks post-exposure when very few snails were found to shed out cercariae. Such information is of practical significance in maintaining colonies of infected *O. hupensis* in the laboratory for production of cercariae or for other research purposes. The results of this study also showed that the rate of infection of *O. hupensis* increased with increased number of introduced miracidia. These results are in agreement with the findings of Ahmed (18), but contrary to the findings of Brooks (19), who reported that when the snail host was exposed to a large number of miracidia, only a very few developed into sporocysts.

The present study showed that the liberation pattern of *S. japonicum* cercaria from *O. hupemsis* (both naturally-infected collected from Dongting Lake, Hunan Province, and laboratory-infected) was of a circadian type. The cercariae started emerging around 07:00 am or one hour after the onset of light in the laboratory, reaching a single peak between 10:00 am and 12:00 pm, and thereafter declining gradually to a minimum at around 18:00 pm. A previous study from Mainland China reporting on emergence of *S. japonicum* cercariae, showed a diurnal pattern that could occur during the periods of the high light intensity, with peak of emergence occurring between 14:00 pm and 17:00 pm (20). Elsewhere, in the Philippines, it was reported that the maximum emergence of *S. japonicum* cercariae from *Oncomelania hupensis quadrasi* occurred during 18:00 pm to 20:00 pm (21). Numerous previous reports have documented that the cercarial emergence coincides with the time when the host snails located downstream are more likely to be in the water (18, 22, 23). The cercariae of *S. mansoni* tend to emerge around noon time when humans are most likely to be in the water. The cercaria of *S. margrebowiei* has two emergence peaks (dawn and dusk) and these peaks coincide with the visit of their vertebrate hosts (antelopes and waterbucks) to the water (24); whereas the emergence of cercariae of *S. rodhaini* is nocturnal, coinciding with the visit of their vertebrate nocturnal host (rodents) to the water (25). The phenomenon of cercarial shedding from snail host may vary from one species to another due to the genetical and geographical variations (26). However, precise information concerning the time of peak emergence of cercariae from the snail host(s) should be helpful for the vertebrate hosts (particularly humans) in terms of avoiding contact with water bodies in schistosomiasis endemic areas, and thus reducing the risk of infection. The present study showed that light as an environmental factor has a positive relationship with shedding of cercariae and rhythmicity of shedding. For example, light from an electric lamp (10 watt) had a quick impact in stimulating liberation of cercariae in relatively large numbers at 2 hours post-exposure to the light, compared to shedding of cercariae under normal daylight. In darkness, very few cercariae were released and did not exhibit any rhythmic pattern; however, it has been reported that cercariae can be shed in small numbers in the dark, but periodic peaks of the output apparently occur because of intrinsic rhythms (27). It has also been previously reported that the cercariae of *O. viverrini* cannot be shed in the dark (28). There are a number of environmental factors that may influence emergence of cercariae from the intermediate hosts, such as, photoperiod, thermo period, pH, dissolved oxygen content of the medium, nutritional conditions, and physiological state of the host; among these light has been thought to act by increasing the snail's body temperature, which probably results in cercarial release (29), as many trematode species cercariae display increased emergence in response to increased water temperature (30, 31).

## Conclusion

In order to clearly understand the parasitic transmission, further research on the subject is deemed necessary, including biotic and abiotic factors that may contribute in determining the effects of *S. japonicum* or other trematode larvae upon *O. hupensis*.

## References

[1] Steinmann P, Keiser J, Bos R, Tanner M, Utzinger J (2006). Schistosomiasis and water resources development: systematic review, meta-analysis, and estimates of people at risk. Lancet Infect Dis..

[2] World Health Organization (2010). Schistosomiasis. http://www.who.int/mediacentre/facesheets/fs115/en/index.html.

[3] World Health Organization (1993). The control of Schistosomiasis.

[4] Jourdane J, Théron A, Rollinson D, Simpson AJG (1987). Larval development: eggs to cercariae. The biology of schistosomes: from genes to latrines.

[5] Xu B, Gong P, Seto E, Liang S, Yang C, Wen S, Qiu D, Gu X, Spear R (2006). A Spatial-Temporal Model for Assessing the Effects of Intervillage Connectivity in Schistosomiasis Transmission. Ann Assoc Am Geogr..

[6] Gerard C, Theron A (1996). Altered nutrition and assimilation of the snail host (*Biomphalaria glabrata*) as a consequence of the parasitic spatial constraint (*Schistosoma mansoni*). Acta Trop..

[7] Brockerhoff AM (2004). Occurrence of the internal parasite *Portunion* sp. (Isopoda: Entoniscidae) and its effect on reproduction in intertidal crabs (Decapoda: Grapsidae) from New Zealand. J Parasitol..

[8] Niemann GM, Lewis FA (1990). *Schistosoma mansoni*: Influence of *Biomphalaria glabrata* size on susceptibility to infection and resultant cercarial production. Exp Parasitol..

[9] Barbosa FS, Coimbra-Junior CE (1992). Alternative approaches in Schistosomiasis control. Mem-Inst-Oswaldo-Cruz..

[10] Mouahid A, Coombes C (1987). Genetic variability of *Schistosoma bovis* cercarial production according to miracidial dose. J Helminthol..

[11] Jordan P, Webbe G (1982). Schistosomiasis epidemiology, treatment and control.

[12] SPSS (2007).

[13] Gerard C (1997). Importance do parasitisme dans la communaute de Gasteropodes de l'Etang de Combourg (Bretagne, France). Parasite..

[14] Gerard C (2001). Structure and temporal variation of trematode parasite and gastropod communities in freshwater ecosystem. Parasite..

[15] Morley NJ, Irwin S, Lewis JW (2003). Pollution toxicity to the transmission of larval digeneans through their molluscan hosts. Parasitology..

[16] Azevedo CM, Borges CC, Andrade ZA (2004). Behavior of *Schistosoma mansoni*-induced his-topathological lesions in *Biomphalaria glabrata* submitted to ionizing radiation. Rev Soc Bras Med Trop..

[17] Tang CT, Lu MK, Guo Y, Wang YN, Peng JY, Wu WB, Li WH, Weimer BC, Chen D (2009). Development of larval *Schistosoma japonicum* blocked in *Oncomelania hupensis* by pre-infection with larval *Exorchis* sp. J Parasitol..

[18] Ahmed AA (1998). Epidemiology of *Schistosoma mansoni* infection in Guneid sugar cane scheme, Gezira state.

[19] Brooks CP (1953). A comparative study of *Schistosoma mansoni* in and *Australorbis glabratus*. J Parasitol..

[20] Théron A, Xia M (1986). Shedding pattern of *Schistosoma japonicum* cercariae from PR China by *Oncomelania hupensis*. Ann Parasitol Hum Comp..

[21] Pesigan TP, Hairston NG, Jauregui JJ, Garcia EG, Santos AT, Santos BC, Besa AA (1958). Studies on *Schistosoma japonicum* infection in the Philippines.2.The molluscan host. Bull. World Health Organ..

[22] Combes C, Théron A (1977). Rhythmes d'emergences des cercaires de trematodes et leur interet dans l'infestation de l'homme et des animaux.

[23] Théron A (1984). Early and late shedding patterns of *Schistosoma mansoni* cercariae: ecological significance in transmission to human and murine hosts. J Parasitol..

[24] Raymond K, Probert AJ (1991). The daily cercarial emission rhythm of *Schistosoma margrebowiei* with particular reference to dark period stimuli. J Helminthol..

[25] Combes C, Fournier A, Moné H, Théron A (1994). Behaviours in trematode cercariae that enhance parasite transmission: patterns and processes. Parasitology..

[26] Kendall SB (1965). Relationships between the species of *Fasciola* and their molluscan hosts. Adv Parasitol..

[27] McClelland WF (1965). Studies on snail vectors of schistosomiasis in Kenya. J Trop Med Hyg..

[28] Kaewkes S, Kaewkes W, Boonmars T, Sripa B (2012). Effect of light intensity on *Opisthorchis viverrini* cercarial shedding levels from *Bithynia* snails--a preliminary study. Parasitol Int..

[29] Asch HL (1972). Rhythmic emergence of *Schistosoma mansoni* cercariae from *Biomphalaria glabrata*: control on illumination. Exp Parasitol..

[30] Mouritsen KN (2002). The Hydrobiaulvae-Maritrema subdolum association: influence of temperature, salinity, light, water pressure and secondary host exudates on cercarial emergence and longevity. J Helminthol..

[31] Poulin R (2006). Global warming and temperature-mediated increases in cercarial emergence in trematode parasites. Parasitology..

